# Prenatal exposure to phenols and benzophenones in relation to markers of male reproductive function in adulthood

**DOI:** 10.3389/fendo.2022.1071761

**Published:** 2022-12-09

**Authors:** Stine A. Holmboe, Louise Scheutz Henriksen, Hanne Frederiksen, Anna-Maria Andersson, Lærke Priskorn, Niels Jørgensen, Anders Juul, Jorma Toppari, Niels E. Skakkebæk, Katharina M. Main

**Affiliations:** ^1^ Department of Growth and Reproduction, Rigshospitalet, Copenhagen, Denmark; ^2^ International Center for Research and Research Training in Endocrine Disruption of Male Reproduction and Child Health (EDMaRC), Copenhagen University Hospital - Rigshospitalet, Copenhagen, Denmark; ^3^ Department of Clinical Medicine, University of Copenhagen, Copenhagen, Denmark; ^4^ Research Centre for Integrative Physiology and Pharmacology, Institute of Biomedicine, University of Turku, Turku, Finland; ^5^ Centre for Population Health Research, University of Turku and Turku University Hospital, Turku, Finland; ^6^ Department of Paediatrics, Turku University Hospital, Turku, Finland

**Keywords:** male reproductive health, reproductive hormones, prenatal exposure, bisphenol A, benzophenone-3, endocrine disruption

## Abstract

**Introduction:**

Environmental exposure during fetal life may disrupt testicular development. In humans, a limited number of studies have investigated whether these adverse effects persist into adulthood. Using data from a prospective, population-based birth cohort study, The Copenhagen Mother-Child cohort, the objective was to assess if there is an association between fetal exposure to selected phenols and benzophenones and markers of testicular function in adult men.

**Methods:**

Pregnant women were recruited in 1997–2001. Their sons were examined clinically at 18-20 years of age, with focus on adult markers of reproductive function (anogenital distance (AGD), semen quality and reproductive hormones). In total, 101 18–20-year-old men were included, whose mothers during pregnancy had a serum sample drawn and analyzed for bisphenol A (BPA) and seven other simple phenols, as well as six benzophenones. To investigate the association between chemical levels (in tertiles, T1-T3) in relation to markers of reproductive function, univariate and multiple linear regression analyses were performed.

**Results:**

In fully adjusted analyses, increased levels of luteinizing hormone (LH) were observed with higher fetal exposure to BPA (percentage difference (95%CI)) (T2: 12% (-8%,36%) and T3: 33% (10%,62%), compared to T1) and benzophenone-3 (BP-3) (T2: 21% (-2%,49%), T3: 18% (-4%,45%)), while no clear association was seen to total testosterone (TT). Higher levels of BPA and BP-3 were associated with a lower TT/LH ratio, although only significant for BPA (p-trend=0.01). No associations were seen to AGD or markers of semen quality.

**Conclusion:**

In conclusion, high exposure to BPA and BP-3 was associated with a compensated reduced Leydig cell function but no other changes in markers of reproductive health. As maternal levels of BPA and BP-3 were not correlated, separate effects may be at play. Larger studies on long-term reproductive consequences of prenatal exposures are warranted to validate our findings.

## 1 Introduction

During recent decades there has been an increase in the incidence of several male reproductive disorders and an increased focus on environmental exposures that may explain some of these trends (1). It has also been suggested that cryptorchidism, hypospadias, poor semen quality and testicular germ cell cancer may be associated through a common etiology involving abnormal fetal development of the male gonads (2–4). In modern societies humans are exposed to numerous industrial chemicals, some of which have documented endocrine disrupting abilities in wildlife and animal studies (5). Examples of such endocrine disrupting pollutants with wide-spread use are phenolic substances as well as benzophenones which are characterized as non-persistent or partly non-persistent because of their short half-lives within the body (6). Fetal life is a critical period for normal development of the male reproductive organs and thus exposure of pregnant women to such chemicals is of concern.

Among the phenols is a group of industrial chemicals which primarily are used as precursors for various types of plastics. This includes bisphenol A (BPA), the most commonly used of the phenols, which is mainly used in polycarbonates and epoxy resins used in some food and drink packaging and also in various electronic equipment, and toys (7). Other phenols, like triclosan (TCS), are known for their antibacterial properties and are commonly used in a range of personal care products (8). Humans are ubiquitously exposed to numerous phenols (9, 10). Besides being detectable in human maternal serum, BPA is also detectable in fetal serum and placenta (11). However, BPA levels in umbilical cord blood are half of those in maternal and placental samples, suggesting some protection by the placenta (12). Studies in rodents have shown that pre- or perinatal exposure to BPA is associated with impaired testis function in offspring, i.e., disrupted spermatogenesis and reduced testosterone level (13–15). However, findings in humans from longitudinal pregnancy cohorts are scarce and the majority is characterized by short follow-up. Some, but not all studies have indicated that prenatal exposures to either BPA or TCS are associated with reduced anogenital distance (AGD), a marker of prenatal androgen action in early life (16–18). To our knowledge, only one study has previously investigated the impact of BPA exposure in relation to markers of reproductive function in adulthood but did not see any clear association (19).

Because of the ultraviolet (UV) absorbing properties, benzophenones are used in a wide range of consumer products, including sunscreen formulations and skin lotions, but also as additives in e.g. plastic food packaging materials, paint, and textiles. In recent years, one of the most used UV absorbers, benzophenone-3 (BP-3) has been detected in most children and adults in US and Danish populations (20–23). Besides being present in pregnant women, several benzophenones have been shown to reach cord blood and fetal blood, although concentrations are lower than in maternal circulation (24). *In vitro* and *in vivo* studies on different species have indicated that some UV filters, e.g. BP-3 can interfere with reproductive hormones by exerting estrogenic or antiandrogenic effects (25–27). In humans, studies on benzophenones in relation to adult male reproductive outcomes are scarce and only include cross-sectional studies of young men rather than studies of *in utero* exposures (28, 29).

In general, human studies on prenatal exposure to environmental chemicals and male reproductive health in adulthood are scarce due to the inherent challenges in performing longitudinal studies with sufficient follow-up time while maintaining an adequate study participation rate. Thus, based on a unique mother-child cohort initiated in 1997 with repetitively, and long-term follow-up of the individuals, the aim in the present study was to investigate the association between maternal serum concentrations of phenols and benzophenones in pregnancy and reproductive function in the adult sons.

## 2 Material and methods

### 2.1 Study population

**Figure 1:**
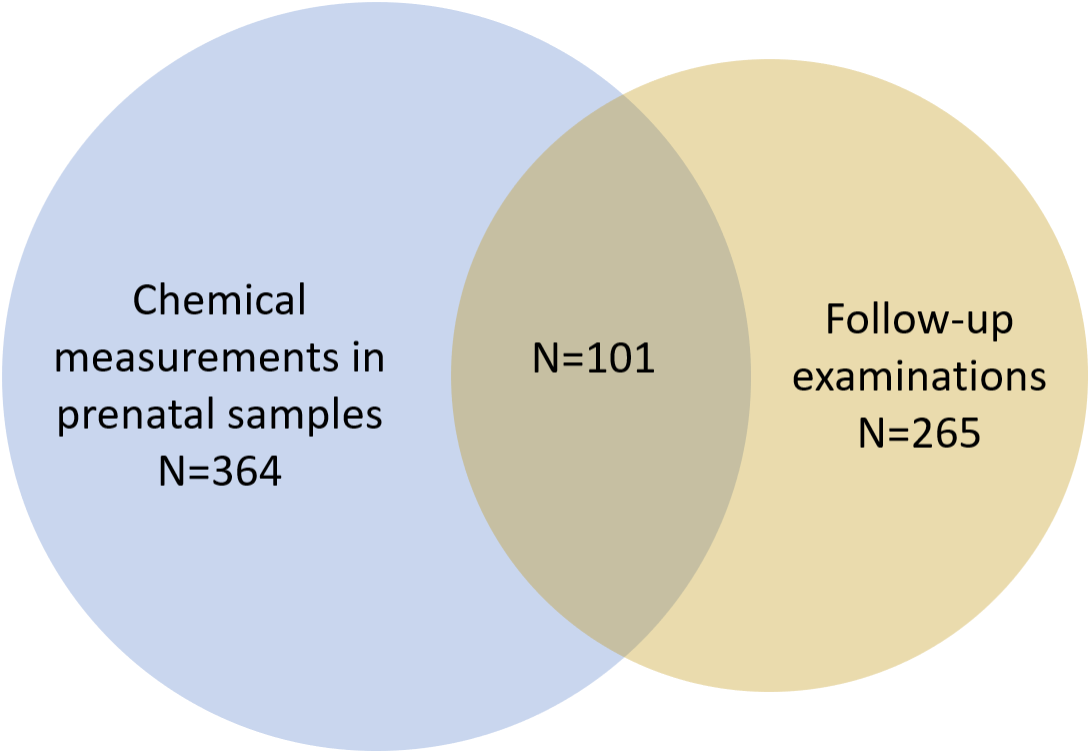
Overview of number of chemical measurements in prenatal samples and number of sons with a follow-up examination.

This study is part of a prospective longitudinal birth cohort study of pregnant women and their sons born between 1997 and 2001 at three hospitals in the Copenhagen area, Denmark. Pregnant women belonging to the referral areas were enrolled during the first trimester of pregnancy and a blood sample was drawn at least once during pregnancy (30), although not all women had material left for biobanking that could be used in the present study. More than 1,000 live-born boys were included in the study with repetitive examinations and when they turned 18 years, they were all invited to participate in an adult follow-up examination at the Department of Growth and Reproduction, Rigshospitalet (31). Men from this adult follow-up examination were included in the present study if a maternal serum sample from pregnancy was available for chemical analyses. In total, 101 young men were included in the study, including a pair of twins (**Figure 1**).

The study was performed following the Declaration of Helsinki II and approved by the Regional Committee on Health Research Ethics ((KF) 01-030/97 and H-17011468) and the Danish Data Protection Agency (1997-1200-074 and VD-2018-118/i-Suite 6358). All participants gave written informed consent prior to participation in the study.

### 2.2 Maternal and birth characteristics

Information on maternal age at birth, smoking habits, parity and birth weight was obtained from hospital records. Pre-pregnancy weight was obtained from self-administered questionnaires during pregnancy.

### 2.3 Follow-up examination of adult sons

All participating young men completed a detailed questionnaire, including information on lifestyle and general health, prior to the day of study participation, at which they delivered a semen sample, had a blood sample drawn for assessment of reproductive hormone levels, and had a physical examination (31). The examination included recording of the men’s weight and height, and BMI was calculated (kg/m^2^). Furthermore, anogenital distance (AGD) was determined; Anoscrotal distance (AGD_AS_, from the posterior base of the scrotum to the anterior wall of the anus) and anopenile distance (AGD_AP_, from the anterior base of the penis to the anterior wall of the anus) were measured with a calliper with the men in lithotomy position. Body fat percentage was assessed by use of a whole-body dual x-ray absorptiometry (DXA) scan (Lunar Prodigy, GE Healthcare, Madison, WI). Participants were compensated for their time (DKK 500 ≈ USD 78).

### 2.4 Chemical analyses of maternal serum samples

In total, serum samples from 364 pregnant women, of which 100 samples belonged to a mother whose son(s) participated in the follow-up examination, were identified and analysed for chemicals at the Department of Growth and Reproduction, Rigshospitalet (**Figure 1**). Samples were analysed for the total concentration (free+conjugated) of 16 chemicals using isotope dilution online Turboflow-liquid chromatography-tandem mass spectrometry (LC-MS/MS) systems equipped with heated electro spray ionization sources changing from positive to negative mode depending on the specific analytes. Prior to the chemical analysis all samples and control materials were enzymatically deconjugated as previously described (22, 24, 32). The chemicals included BPA, TCS, triclocarban (TCC), three chlorinated phenols, two phenyl phenols, and six benzophenones (for full chemical names, abbreviations, and limits of detection (LOD) see **Table 1**). In brief, all maternal serum samples were analyzed for the benzophenones in 13 batches and for the phenols in 14 batches, all including standards for calibration curves, two blanks, three un-spiked serum pool controls, and two times three serum pool controls spiked with a mixture of native benzophenone and phenol standards in low (QC_low_) and high (QC_high_) concentration levels. The mean recovery was > 82% for all analytes except QC_low_ for BP-7 (73%) and 4-PP (74%). The relative standard deviations (RSD) ranged from 7-15% in both spike levels for all analytes except QC_low_ for BP-7 (19%), BPA (23%), TCC (26%) and 4-PP (18%) and QC_high_ for TCC (24%).

**Table 1:** Chemical names, abbreviations, LODs (limit of detection) and serum concentrations of phenols and benzophenones in 100 prenatal serum samples (percentiles).

Group	Abbreviation		LOD (ng/mL)		% > LOD									
				10	25	50	75	90	Max
Phenols														
Bisphenol A	BPA	0.18		95		0.3	0.7		1.7		2.3		2.9	21.0
Triclosan	TCS	0.22		99		1.1	2.0		5.3		19.3		34.9	49.7
Triclocarban*	TCC	0.27		0										<LOD
2,4-dichlorophenol*	2,4-DCP	0.18		15							<LOD		0.3	1.8
2,5-dichlorophenol*	2,5-DCP	0.11		5									<LOD	0.4
2,4,5-trichlorophenol*	2,4,5-TCP	0.29		3									<LOD	0.6
2-phenylphenol	2-PP	0.13		78		<LOD	0.4		0.5		0.7		0.9	1.3
4-phenylphenol*	4-PP	0.12		4									<LOD	0.6
UV filters														
Benzophenone-1*	BP-1	0.13		6									<LOD	2.1
Benzophenone-2*	BP-2	0.08		11							<LOD		0.2	0.6
Benzophenone-3	BP-3	0.12		100		0.4	0.7		0.9		1.3		1.7	22.2
5-cholro-2-hydroxybenzophenone	BP-7	0.24		26					<LOD		0.3		0.6	1.4
4-hydroxy-benzophenone	4-HBP	0.18		100		1.7	2.2		2.9		3.6		4.1	5.9
4-methyl-benzophenone	4-MBP	0.27		100		2.3	2.8		3.9		5.1		6.0	8.2

*Chemicals were detectable in < 30 samples and not included in further analyses

### 2.5 Hormone analyses of serum samples from adult sons

All men had a fasting blood sample drawn in the morning. Samples were centrifuged and stored at -20°C until analysis. All serum hormone measurements were performed in the same laboratory at the Department of Growth and Reproduction in 2020. Testosterone, estradiol and sex hormone-binding globulin (SHBG) were measured by a chemiluminescent enzyme immunoassay (Access2, Beckman Coulter, Brea, CA, USA), follicle stimulating hormone (FSH) and luteinizing hormone (LH) were measured using a two-sided fluoroimmunometric assay (DELFIA, Wallac Oy, Turku, Finland), inhibin B levels were measured by an enzyme-linked immunosorbent assay (ELISA, Inhibin B gen II, Beckman Coulter). Insulin-like factor 3 (INSL3) was determined using liquid chromatography-mass spectrometry (LC-MS/MS) with a limit of detection (LOD) of 0.03 μg/L (33). Finally, insulin-like growth factor 1 (IGF-1) and insulin-like growth factor-binding protein 3 (IGFBP-3) levels were analyzed by automated chemiluminescence immunoassay (IDS-ISYS, Immunodiagnostic systems, Boldon, United Kingdom). In the full measurement range, the interassay coefficients of variation were less than 21% for INSL3, less than 8% for inhibin B, 7% for testosterone, estradiol, SHBG and IGFBP-3, and less than 5% for IGF-1, LH and FSH. Free testosterone was calculated based on the level of total testosterone and SHBG assuming a fixed albumin level of 43.0 g/L using the equation by Vermeulen et al. (34). Derived ratios between total testosterone and LH (TT/LH) and inhibin B and FSH were calculated.

### 2.6 Semen analyses

All men provided a semen sample by masturbation in a room close to the semen laboratory and samples were kept at 37°C until analysis. The period of ejaculation abstinence was recorded. The men had been asked to abstain from ejaculation for at least 48 h before sampling but were still included if abstinence time was shorter. Semen analyses were performed in agreement with the World Health Organization (WHO) guidelines (35). Briefly, semen volume was determined by weighing, sperm concentration was assessed using an automated sperm cell counter (NucleoCounter^®^ NC-3000™, Chemometec A/S, Alleroed, Denmark) and the total sperm count was calculated as the product of sample volume and sperm concentration. Sperm motility was assessed by placing 10 µl semen on a glass slide and using phase-contrast microscopy. Spermatozoa were classified as either progressive motile, non- progressive motile or immotile. Fixed and Papanicalaou stained morphology slides were prepared and evaluated according to ‘strict criteria’ (36). All assessments were done in duplicates by the same observer and the average was used.

### 2.7 Statistical methods

Measurements for each chemical were presented as percentiles and maximum values as well as number of samples above the analytical LOD (**Table 1**). In further statistical analyses, all non-detectable chemical values (< LOD) were replaced by LOD/√2. To elucidate if mothers of men who participated in the follow-up examination differed from mothers of men who did not, a comparison of prenatal chemical levels for the two groups was performed using a Mann-Whitney U test and of maternal basic characteristics.

Basic characteristics and levels of reproductive hormones (n=101), semen parameters (n=99) and AGD (n=88) for the total study population are presented as medians and inter-quartile range.

For chemicals where less than 30% of the samples had concentrations above LOD, analyses of associations with outcomes were not performed. For the remaining chemicals, the marginal correlation between each of the compounds were tested using Spearman’s rank-order correlation. Associations between chemical levels in relation to reproductive hormones, semen parameters and AGD were performed using univariate and multiple linear regression analysis. To accommodate potential nonlinear associations, chemical levels were analyzed in tertiles (T1-T3) and a trend test was performed by inserting the tertiles in the model as a continuous variable.

To obtain normally distributed residuals and homoscedasticity, reproductive hormones and the two measures of AGD were transformed by natural logarithm (ln). Testicular volume, sperm concentration and total sperm count were transformed by ln whereas the variables “% morphological normal sperm” and “% progressive motile sperm” remained untransformed. Covariates were selected *a priori* based on literature and included potential confounders for the association as well as covariates closely related to variation in the exposure or outcome measurements (i.e., time of blood sampling and period of ejaculation abstinence). Models were adjusted stepwise; an unadjusted model of the marginal association between exposure and outcome, Model I including adjustment for maternal factors (maternal smoking (yes/no), gestational age at blood sampling (continuous variable)), and Model II including additional adjustment for covariates related to the son (smoking status of son (daily smoker/occasional smoker/non-smoker), body fat percentage (continuous variable), weekly alcohol intake (continuous variable) and physical fitness (Good or very good/moderate/poor). All adjusted analyses with reproductive hormones were further adjusted for time of blood sampling (continuous variable), adjusted analyses with AGD measurements for height of the son, adjusted analyses with semen parameters for period of abstinence (continuous variable) and adjusted analyses with sperm motility for the time between delivery of semen sample and start of motility assessment (continuous variable). Estimates based on ln-transformed outcome variables were back-transformed using the formula: 100*(exp(β)-1), as described in (37). to reflect the percentage differences in outcome variables according to tertiles of chemical levels.

In subanalyses, further adjustment for maternal age (continuous variable) and parity (1, 2, 3 or more) was performed in separate models. Also, one man with azoospermia was excluded from analyses with semen parameters as the outcome, and the findings were compared to the main findings. Finally, to account for multiple testing, all p-values from fully adjusted models were adjusted according to the Benjamini-Hochberg (38, 39) method and compared with the p-values without this adjustment.

P-values < 0.05 were considered statistically significant. Analyses were performed using SPSS v. 25 (Armonk, NY: IBM Corp, USA).

## 3 Results

Of the included chemicals, only BPA, TCS, 2-phenylphenol, BP-3, 4-hydroxy-benzophenone (4-HBP) and 4-methyl-benzophenone (4-MBP) could be detected in more than 30% of the samples (**Table 1**). BPA showed a significant weak correlation with 2- phenylphenol (r=0.20, p=0.04) whereas BP-3 was moderately correlated to 2-phenylphenol (r=0.33, p<0.01), 4-HBP (r=0.55, p<0.01) and 4-MBP (r=0.34, p<0.01). Finally, 2-phenylphenol and 4-MBP were moderately correlated (r=0.41, p<0.01) whereas no other significant correlations were observed (data not shown).

**Figure 2:**
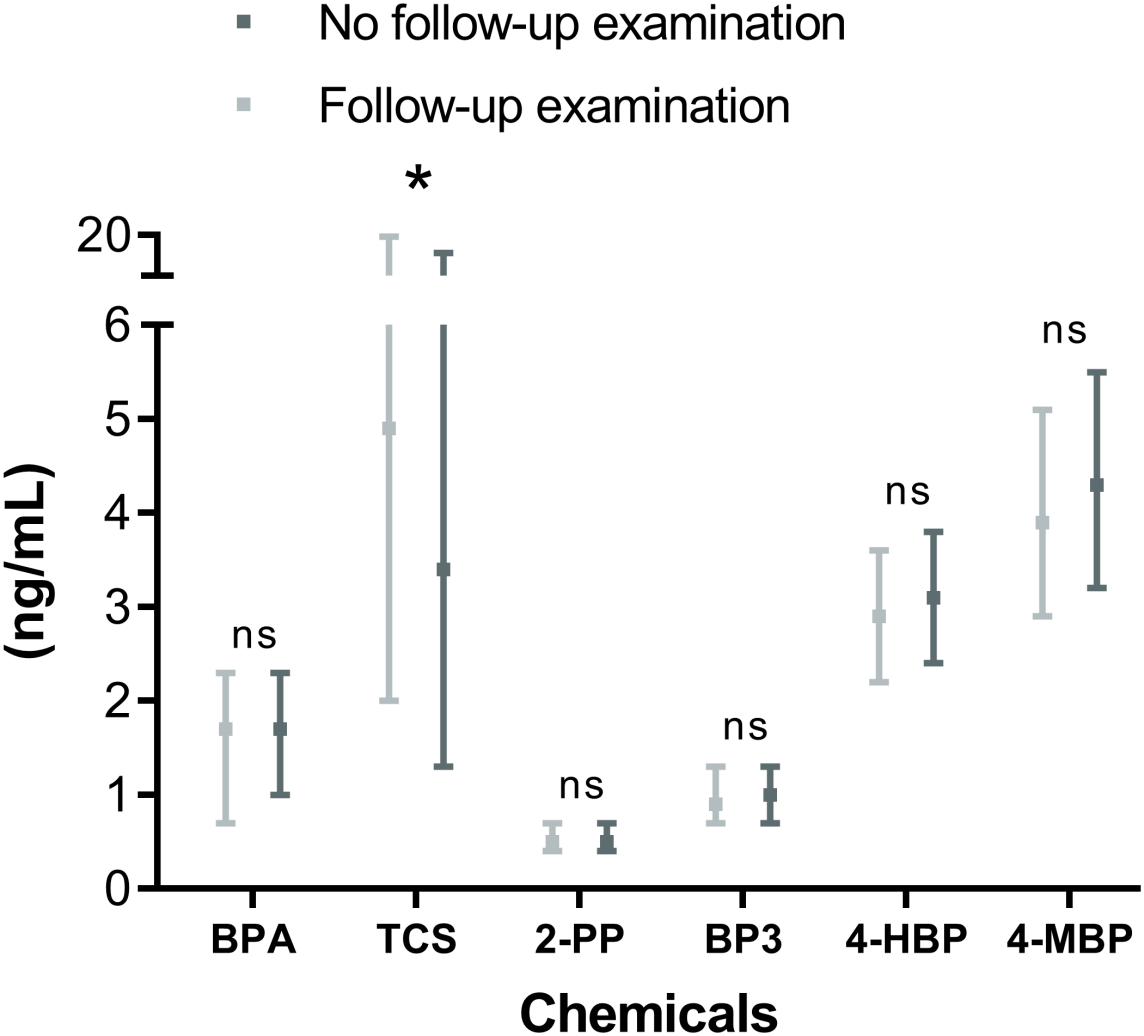
Comparison of chemical levels in pregnancy serum samples from mothers with sons with (n=100) or without (n=263) a follow-up examination. P-values <0.05 are indicated by an asterisk. BPA, bisphenol A; TCS, triclosan; 2-PP, 2-phenylphenol; BP-3, benzophenone-3; 4-HBP, 4-hydroxy-benzophenone; 4-MBP, 4-methyl-benzophenone.

No difference in chemical concentrations was observed between mothers of men who did not participate in the follow-up examination and men who did except for TCS (**Figure 2**). Median TCS levels were significantly higher in mothers of men who participated in the follow-up (5.3 ng/mL vs 3.4 ng/mL, respectively, p=0.015).

The mothers’ median age at delivery was 31 years and their sons were 19 years old at follow-up (**Table 2**). Their median sperm concentration was 52 million/ml and the median total sperm count was 131 million (**Table 3**).

**Table 2:** Basic characteristics of the study population.

	Follow-up of son	No follow-up of son
	Median (25-75 pct) or % (n)	
Maternal factors		
Maternal factors N	100	264
Maternal age at delivery (years)	31.1 (28.3-34.1)	31.0 (28.0-33.9)
Pre-pregnancy weight (kg)	62.5 (58.4-67.8)	64.5 (58.6-72.0)
Maternal smokers (%)	23.0 (23)	22.7 (60)
Information adult son		
N	101	
Birth weight (kg)	3.6 (3.2-4.0)	
Age (years)	19.3 (18.9-19.7)	
Weight (kg)	72.5 (65.5-81.1)	
Height (cm)	184.5 (179.8-189.7)	
BMI (kg/m^2^)	21.2 (19.7-23.5)	
Body fat %	19.3 (16.7-24.0)	
Smoking frequency		
Non-smoker	48 (48)	
Occasional smoker	42 (42)	
Daily smoker	11 (11)	
Weekly alcohol intake	1 (0-10)	
Good physical fitness*	55.0 (55)	

*Good or very good self-rated physical fitness

**Table 3:** Description of outcome variables for the study population with maternal chemical measurements and follow-up examination (n=101).

	N	Median (25-75 pct)
Hormones		
Total testosterone (nmol/L)	101	20.1 (17.5-24.4)
SHBG (nmol/L)	101	33.6 (27.7-41.4)
Free Testosterone (pmol/L)	101	452 (383-514)
Estradiol (pmol/L)	101	93 (81-111)
LH (IU/L)	101	4.0 (3.0-5.2)
TT/LH ratio	101	5.4 (3.8-6.8)
INSL3 (mg/L)	101	1.3 (1.1-1.7)
FSH (IU/L)	101	3.0 (2.1-5.2)
Inhibin B (pg/ml)	101	186 (143-239)
Inhibin B/FSH ratio	101	69 (32-107)
IGF-1 (ng/ml)	101	278 (249-326)
IGFBP3 (ng/ml)	101	4397 (4067-4879)
Time of blood sampling (hh:mm)	101	9:25 (8:50-10:29)
Semen parameters		
Semen volume (ml)	99	3.0 (2.0-4.1)
Sperm concentration (million/ml)	99	52 (22-81)
Total sperm count (million)	99	131 (56-284)
Progressively motile spermatozoa (%)	99	60 (47-70)
Morphologically normal spermatozoa (%)	99	8 (5-11)
Ejaculation abstinence (h)	99	62 (48-85)
Time until motility analysis (min)	92	46 (40-51)
Anogenital distance		
AGD_AS_ (cm)	88	5.9 (5.0-7.1)
AGD_AP_ (cm)	88	12.7 (12.0-13.5)

**Figure 3:**
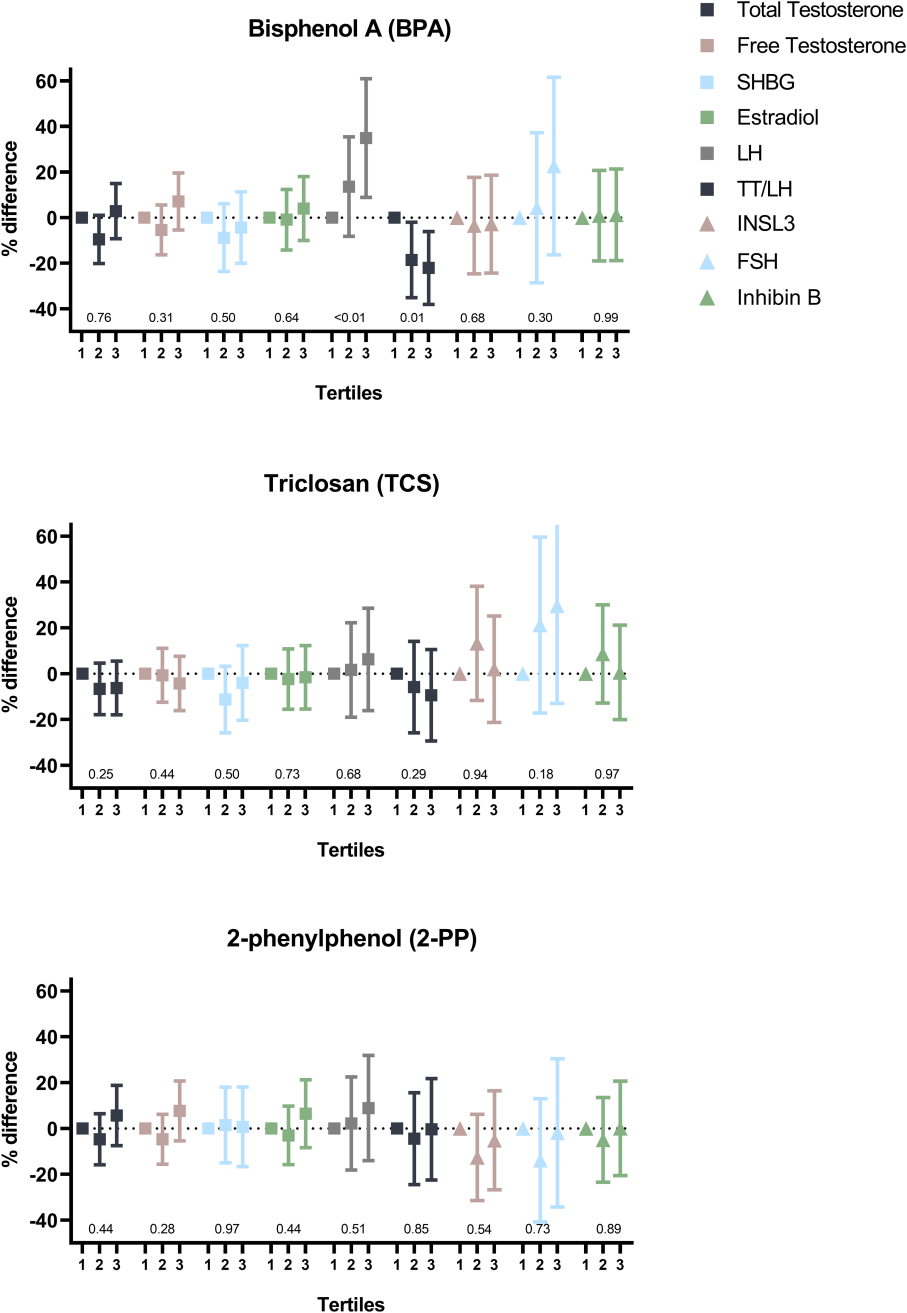
Percentage difference in levels of serum hormone concentrations according to tertiles of prenatal phenol exposure levels adjusted for confounders. P-values indicate trend across categories. SHBG, sex hormone-binding globulin; LH, luteinizing hormone; TT/LH, total testosterone/Luteinizing hormone ratio; INSL3, Insulin-like factor 3; FSH, follicle stimulating hormone.

**Figure 4:**
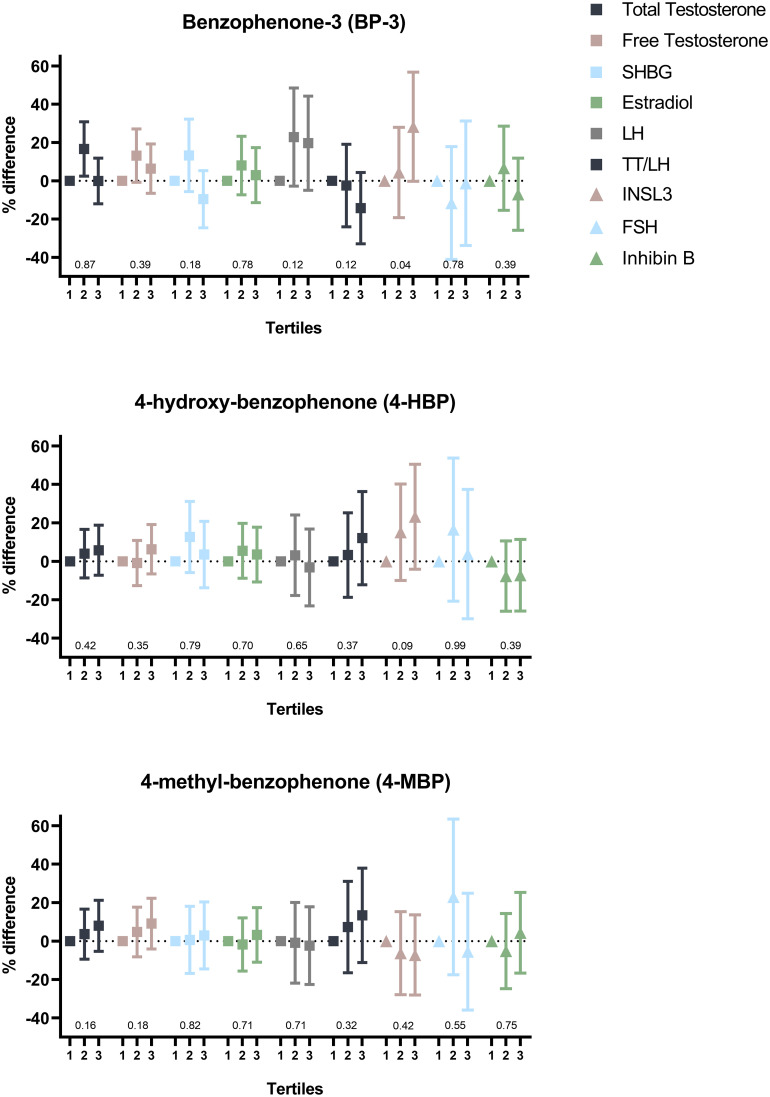
Percentage difference in levels of serum hormone concentrations according to tertiles of prenatal benzophenone exposure level adjusted for confounders. P-values indicate trend across categories. SHBG, sex hormone-binding globulin; LH, luteinizing hormone; TT/LH, total testosterone/Luteinizing hormone ratio; INSL3, Insulin-like factor 3; FSH, follicle stimulating hormone.

In fully adjusted analyses, higher prenatal levels of BPA (evaluated in tertiles, T1-T3) were significantly associated with higher LH levels in sons (T2: 12% (-8%,36%) and T3: 33% (10%,62%) compared to the reference category, T1, respectively) (**Figure 3**; **Supplementary Tables 1**–**3**) but not with total testosterone. Hence, higher BPA levels were associated to lower TT/LH ratio (T2: -20% (-35%,-1%), T3: -23% (-38%,-6%). An inverted U-shaped association was seen for BP-3 exposure and total testosterone level (T2: 16% (3%,31%), T3: -1% (-12%,12%)) and to a lesser degree for free testosterone (T2: 13% (-1%,27%) and T3: 6% (-6%,19%) compared to T1) (**Figure 4**; **Supplementary Tables 4–6**). Also, a positive association between BP-3 exposure and LH levels was seen (T2: 21% (-2%,49%), T3: 18% (-4%,45%) compared to T1). Finally, a tendency of higher INSL3 levels with higher tertile of BP-3 was seen (p-trend = 0.04). For 4-HBP a tendency was seen of higher exposure levels being associated to higher levels of INSL3 (p-trend = 0.09) as well as lower levels of IGF-1 (p-trend = 0.06). For prenatal TCS exposure, no significant associations were found. However, a tendency of higher levels of FSH with higher TCS exposure was seen. No clear associations were seen for 2-phenylphenol in relation to the observed outcomes.

No clear associations were seen between any of the chemicals and the semen parameters and AGD with exception of 2-phenylphenol that tended to be positively associated to higher sperm concentration and total sperm count (p-trend = 0.17 and p-trend = 0.11, respectively) (**Supplementary Tables 7–12**).

In subanalyses, further adjustment for maternal age or parity did not alter the observed findings. In general, excluding the one azoospermic man from analyses did not change the results. After adjusting for multiplicity, no associations remained statistically significant (data not shown).

## 4 Discussion

In this long-term prospective study, we observed that young men prenatally exposed to the highest levels of BPA and BP-3 had higher LH levels in adulthood and a lower TT/LH ratio indicating a compensated reduced Leydig cell function. However, no associations were seen to AGD as a marker of prenatal androgen action or to markers of semen quality. Testosterone production in men is characterized by a buffer capacity so that testosterone levels to some degree can be sustained by a compensatory increase in LH stimulation. Although our observational study cannot prove causality, our findings indicate a long-lasting effect of prenatal BPA and BP-3 exposure on Leydig cell function which has been described in experimental studies. A compensated Leydig cell function has been associated with an increased all-cause mortality in a long term follow-up study (40). Thus, we speculate that the observed compensated Leydig cell function in this cohort of young men may become clinically relevant over time and reflect a potential health risk.

Several *in vitro* studies have shown that BPA has both estrogenic and anti-androgenic properties due to its capability to bind and activate the estrogen receptor although with much lower affinity than estradiol (41, 42) as well as the androgen receptor (43). In addition, BPA can inhibit steroidogenesis *via* a number of pathways in both human and rodent testis *in vitro* and thus, exert antiandrogenic effects (44, 45). Rodent studies of postnatal effects of *in utero* exposure to BPA are conflicting and differences in findings are likely explained by variations in doses and duration of exposure as well as differences between species. However, prenatal and perinatal BPA exposure has in the majority of studies been associated to reduced testis weight and testosterone level as well as a reduction in key enzymes and proteins in steroidogenesis in male offspring, indicating a disruption of Leydig cell function [reviewed in (46)].

Anogenital distance (AGD) is the distance from anus to the genitals and has in rodents been shown to be determined by androgen action in early fetal life (47). Thus, reduced AGD may be indicative of insufficient testosterone during the early stages of reproductive development. Furthermore, in adult men a shorter AGD has been associated with impaired semen quality (48). Few human studies have investigated the association between prenatal BPA exposure and AGD with conflicting results. Several studies found no association between maternal BPA levels and AGD levels in newborns (17, 18, 49) in line with our findings, whereas one study observed that higher maternal BPA levels were associated with shorter AGD among 12-month-old boys (18). Notably, in a real-life scenario, exposure to BPA occurs continuously throughout pregnancy and postnatally, which complicates human observational studies compared to controlled exposure settings in rodents and *in vitro* experiments.

To our knowledge, only one other longitudinal pregnancy cohort has investigated the association between prenatal BPA levels and reproductive hormones in adulthood. In contrast to our findings, results from that cohort indicated that maternal serum levels of BPA were positively associated with the sons’ sperm concentration and motility in adulthood, whereas no association was seen with levels of reproductive hormones, including LH (19). This difference may be explained by differences in BPA exposure levels which was higher in our cohort compared to the levels observed in the Raine cohort (median (max): 1.7 ng/ml (21 ng/ml) vs. 0.25 ng/ml (10.5 ng/ml), respectively). Cross-sectional studies of young adult men have observed a positive association between urinary BPA levels and LH levels in line with our findings (50, 51). However, one of the studies also observed that higher urinary levels of BPA were associated with higher levels of total testosterone and estradiol suggesting that BPA in adult men could act as a partial antagonist to both the estrogen and androgen receptor (50).

In the present study, higher levels of BP-3 were associated with higher LH levels and consequently a lower TT/LH ratio whereas an inverted U-shaped association was seen in relation to testosterone levels. Maternal levels of BPA and BP-3 were not correlated, indicating different exposure patterns and thus, separate effects may be at play. An *in vivo* study in rodents showed that male offspring dermally exposed to BP-3 at up to 7 weeks of age and with dermally exposed mothers during pregnancy showed a substantial reduction in plasma testosterone levels (52). This may be explained by a direct adverse effect of BP-3 on the Leydig cells or on steroidogenesis in general. Thus, a decrease in testosterone could subsequently be followed by a compensatory increase in LH in line with our findings. More studies in humans are, however, needed to confirm the associations seen for BP-3 in relation to testosterone and LH, respectively.

To our knowledge, no longitudinal studies in humans have investigated the association between prenatal BP-3 exposure and reproductive hormones in adulthood, however, few cross-sectional studies have been performed with conflicting results. In a cohort of 215 young Spanish men, higher urinary BP-3 concentrations were associated with higher FSH levels, whereas no associations were seen with LH or other reproductive hormones (28). However, in a study of 195 young Danish men, higher urinary BP-3 levels were associated with higher total testosterone and estradiol levels and lower FSH levels but only in men with a loss-of-function mutation in the filaggrin gene, important for normal skin barrier, and thereby probably generally more exposed dermally (29).The levels of BP-3 were in general higher in the Danish men compared to the Spanish men (median levels: 2.8 ng/ml vs. 1.3 ng/ml), and it is possible, that different levels of co-exposures not accounted for in the studies also could contribute to the observed differences in findings.

In contrast to cross-sectional studies, a key advantage of the present study is the use of a prospective birth cohort with information on chemical exposures during a critical period where reproductive development takes place (3) and with detailed follow-up information including a comprehensive andrological examination. Some limitations should, however, be considered when interpreting the results. Multiple factors are known or suspected to affect reproductive outcomes in the young men. This includes adult exposure to phenols and benzophenones as well as exposures from additional EDCs [reviewed in (53)] which was not taken into consideration in this study. We also did not explore simultaneous fetal exposure to other chemicals in this study, although animal studies have shown that mixed exposures to different chemical groups during fetal life may have additive effects on reproductive outcomes (54). Although we adjusted analyses of the association between exposure levels and adult reproductive function for several confounders, we could not control for variations in this over time. Furthermore, we cannot exclude, that our findings are influenced by residual confounding not accounted for in the present study.

Another limitation is the small study size, which limits the statistical power. Furthermore, no associations were significant after adjustment for multiplicity. Thus, we cannot rule out, that our findings are false positive (type I errors) and the data should be replicated in larger studies. This project applied a single prenatal exposure measurement and a single adult reproductive health assessment despite the well-known intra-individual variation in both (55), which increases the risk of exposure and outcome misclassification. However, these would be expected to be random and most likely attenuate any true association. We speculate that the endogenous negative feedback mechanism between LH and testosterone may have been the most sensitive parameter for finding subtle long-term effects of prenatal exposures. In contrast, semen quality shows a high intra-individual variation which could attenuate potential associations (56). Thus, our findings need to be replicated in larger populations. Furthermore, we cannot rule out that genetic factors could play a role for the observed associations which was however, not investigated in the present study. Another limitation is that exposure levels were analyzed in serum samples as maternal urine samples were not available. Urine is considered the preferred matrix to determine exposures of non-persistent compounds due to their short half-lives and subsequent rapid metabolization and excretion into urine (57). Also for bisphenols, urine is the preferred matrix to determine the concentrations (58), although urine alone also has been acknowledged as unsuitable to assess the full toxicokinetics of bisphenols (59). Moderate correlations have furthermore been observed between some benzophenones determined in serum compared to urine (23). Since serum concentrations are lower compared to urine concentrations, a higher proportion of the samples will be below detection level with the risk of underestimating the exposure in the lower range.

In conclusion, our findings indicate that young men prenatally exposed to BP-3 or BPA were characterized by a compensated reduced Leydig cell function in adulthood whereas no association was seen in relation to AGD or semen quality. Larger studies on long-term reproductive consequences of prenatal exposures are warranted to validate our findings.

## Data availability statement

The datasets presented in this article are not readily available because the data are not publicly available due to ethical restrictions. Requests to access the datasets should be directed to Katharina M. Main, Katharina.Main@regionh.dk.

## Ethics statement

The studies involving human participants were reviewed and approved by the Regional Committee on Health Research Ethics ((KF) 01-030/97 and H-17011468) and the Danish Data Protection Agency (1997-1200-074 and VD-2018-118/i-Suite 6358). The patients/participants provided their written informed consent to participate in this study.

## Author contributions

Conceptualization: SH, A-MA, AJ, JT, NS, KM. Data collection: LS, HF, KM. Data analysis and interpretation: SH, LS, A-MA, LP, NJ, NS, KM. All authors had full access to the data in the study and take responsibility for the integrity and the accuracy of the data analysis. All authors critically reviewed the manuscript and approved the submitted version. All authors contributed to the article and approved the submitted version.
