# Supplementary material for: Prenatal exposure to phenols and benzophenones in relation to markers of male reproductive function in adulthood
Source: Front Endocrinol (Lausanne). 2022 Dec 9;13:1071761. doi: 10.3389/fendo.2022.1071761 (PMC9780366; doi:10.3389/fendo.2022.1071761)
Supplement: Supplementary file 1 [file Table_1.docx]

Supplementary Material

| Supplementary table 1: Linear regression analysis of prenatal serum levels of Bisphenol A (tertiles) and reproductive hormones (back-transformed estimates to reflect percentage difference) in adulthood | | | | | | |
| --- | --- | --- | --- | --- | --- | --- |
|  | |  | Prenatal Bisphenol A levels | | |  |
|  | |  | Lowest tertile | Second tertile | Highest tertile | p-trend |
|  | |  |  |  |  |  |
| Total Testosterone | |  |  |  |  |  |
|  | Unadjusted |  | Ref. | -9 (-19, 2) | 3 (-9, 15) | 0.67 |
|  | Model I** |  | Ref. | -10 (-20, 2) | 1 (-10, 14) | 0.81 |
|  | Model II*** |  | Ref. | -10 (-20, 1) | 2 (-9, 15) | 0.76 |
| SHBG | |  |  |  |  |  |
|  | Unadjusted |  | Ref. | -4 (-19, 13) | -4 (-19, 14) | 0.64 |
|  | Model I** |  | Ref. | -7 (-22, 10) | -7 (-22, 10) | 0.38 |
|  | Model II*** |  | Ref. | -10 (-23, 6) | -5 (-20, 12) | 0.50 |
| Free Testosterone | |  |  |  |  |  |
|  | Unadjusted |  | Ref. | -8 (-17, 3) | 6 (-5, 18) | 0.31 |
|  | Model I** |  | Ref. | -7 (-17, 5) | 6 (-5, 19) | 0.28 |
|  | Model II*** |  | Ref. | -6 (-16, 6) | 7 (-5, 20) | 0.31 |
| Estradiol | |  |  |  |  |  |
|  | Unadjusted |  | Ref. | -4 (-15, 8) | 5 (-8, 19) | 0.46 |
|  | Model I** |  | Ref. | -2 (-14, 11) | 6 (-6, 21) | 0.32 |
|  | Model II*** |  | Ref. | -1 (-14, 13) | 3 (-10, 18) | 0.64 |
| LH | |  |  |  |  |  |
|  | Unadjusted |  | Ref. | 6 (-11, 28) | 32* (10, 58) | <0.01 |
|  | Model I** |  | Ref. | 9 (-10, 32) | 35* (12, 63) | <0.01 |
|  | Model II*** |  | Ref. | 12 (-8, 36) | 33* (10, 62) | <0.01 |
| Total Testosterone/LH | |  |  |  |  |  |
|  | Unadjusted |  | Ref. | -14 (-30, 5) | -22* (-36, -5) | 0.01 |
|  | Model I** |  | Ref. | -17 (-33, 2) | -25* (-39, -8) | <0.01 |
|  | Model II*** |  | Ref. | -20* (-35, -1) | -23* (-38, -6) | 0.01 |
| INSL3 | |  |  |  |  |  |
|  | Unadjusted |  | Ref. | -3 (-21, 20) | -5 (-23, 17) | 0.62 |
|  | Model I** |  | Ref. | -3 (-22, 20) | -4 (-22, 18) | 0.68 |
|  | Model II*** |  | Ref. | -5 (-24, 18) | -4 (-24, 19) | 0.68 |
| FSH | |  |  |  |  |  |
|  | Unadjusted |  | Ref. | 3 (-23, 37) | 19 (-11, 58) | 0.23 |
|  | Model I** |  | Ref. | 2 (-25, 38) | 20 (-11, 61) | 0.23 |
|  | Model II*** |  | Ref. | 1 (-27, 39) | 19 (-14, 63) | 0.30 |
| Inhibin B | |  |  |  |  |  |
|  | Unadjusted |  | Ref. | 3 (-15, 24) | 4 (-13, 26) | 0.64 |
|  | Model I** |  | Ref. | -1 (-19, 20) | 1 (-16, 22) | 0.90 |
|  | Model II*** |  | Ref. | 0 (-18, 21) | 0 (-18, 22) | 0.99 |
| IGF-1 | |  |  |  |  |  |
|  | Unadjusted |  | Ref. | 7 (-4, 18) | 6 (-5, 17) | 0.29 |
|  | Model I** |  | Ref. | 5 (-6, 17) | 4 (-6, 16) | 0.45 |
|  | Model II*** |  | Ref. | 5 (-5, 17) | 0 (-10, 11) | 0.96 |
| IGFBP-3 | |  |  |  |  |  |
|  | Unadjusted |  | Ref. | 5 (-2, 13) | 1 (-6, 8) | 0.83 |
|  | Model I** |  | Ref. | 4 (-3, 12) | 0 (-7, 8) | 0.95 |
|  | Model II*** |  | Ref. | 3 (-4, 11) | -2 (-9, 5) | 0.60 |
| *p-value < 0.05, percentage difference was calculated as 100*(exp(β)-1), **adjusted for time of blood sampling (continuous variable), maternal smoking (yes/no), gestational age at blood sampling (continuous variable)), ***further adjusted for smoking status of son (daily smoker/occasional smoker/non-smoker), body fat percentage (continuous variable), weekly alcohol intake (continuous variable) and physical fitness (Good or very good/moderate/poor), Abbreviations: SHBG, sex hormone-binding globulin; LH, luteinizing hormone; INSL3, Insulin-like factor 3; FSH, follicle stimulating hormone; IGF-1, insulin-like growth factor 1; IGFBP-3, insulin-like growth factor-binding protein 3. | | | | | | |

| Supplementary table 2: Linear regression analysis of prenatal serum levels of triclosan (TCS) (tertiles) and reproductive hormones (back-transformed estimates to reflect percentage difference) in adulthood | | | | | | |
| --- | --- | --- | --- | --- | --- | --- |
|  | |  | Prenatal Triclosan (TCS) levels | | |  |
|  | |  | Lowest tertile | Second tertile | Highest tertile | p-trend |
|  | |  |  |  |  |  |
| Total Testosterone | |  |  |  |  |  |
|  | Unadjusted |  | Ref. | -6 (-17, 6) | -5 (-16, 7) | 0.38 |
|  | Model I** |  | Ref. | -7 (-18, 5) | -5 (-16, 7) | 0.38 |
|  | Model II*** |  | Ref. | -10 (-20, 1) | 2 (-9, 15) | 0.25 |
| SHBG | |  |  |  |  |  |
|  | Unadjusted |  | Ref. | -9 (-23, 8) | -2 (-17, 16) | 0.79 |
|  | Model I** |  | Ref. | -9 (-24, 8) | -4 (-19, 14) | 0.58 |
|  | Model II*** |  | Ref. | -12 (-25, 4) | -5 (-20, 13) | 0.50 |
| Free Testosterone | |  |  |  |  |  |
|  | Unadjusted |  | Ref. | -2 (-13, 9) | -4 (-15, 7) | 0.43 |
|  | Model I** |  | Ref. | -3 (-14, 9) | -3 (-14, 9) | 0.57 |
|  | Model II*** |  | Ref. | -1 (-12, 11) | -5 (-16, 8) | 0.44 |
| Estradiol | |  |  |  |  |  |
|  | Unadjusted |  | Ref. | -3 (-15, 10) | -7 (-18, 6) | 0.28 |
|  | Model I** |  | Ref. | -3 (-15, 10) | -5 (-17, 8) | 0.43 |
|  | Model II*** |  | Ref. | -3 (-15, 11) | -2 (-15, 13) | 0.73 |
| LH | |  |  |  |  |  |
|  | Unadjusted |  | Ref. | 1 (-17, 22) | 1 (-16, 23) | 0.90 |
|  | Model I** |  | Ref. | -1 (-19, 21) | 2 (-17, 24) | 0.86 |
|  | Model II*** |  | Ref. | 0 (-18, 23) | 5 (-15, 29) | 0.68 |
| Total Testosterone/LH | |  |  |  |  |  |
|  | Unadjusted |  | Ref. | -7 (-24, 15) | -6 (-24, 15) | 0.53 |
|  | Model I** |  | Ref. | -6 (-24, 17) | -7 (-25, 15) | 0.51 |
|  | Model II*** |  | Ref. | -7 (-25, 15) | -11 (-29, 11) | 0.29 |
| INSL3 | |  |  |  |  |  |
|  | Unadjusted |  | Ref. | 2 (-17, 25) | -3 (-21, 20) | 0.79 |
|  | Model I** |  | Ref. | 6 (-14, 32) | 0 (-19, 24) | 0.99 |
|  | Model II*** |  | Ref. | 11 (-11, 39) | 0 (-20, 26) | 0.94 |
| FSH | |  |  |  |  |  |
|  | Unadjusted |  | Ref. | 15 (-13, 54) | 20 (-10, 60) | 0.21 |
|  | Model I** |  | Ref. | 19 (-12, 61) | 23 (-9, 66) | 0.18 |
|  | Model II*** |  | Ref. | 17 (-15, 61) | 25 (-11, 74) | 0.18 |
| Inhibin B | |  |  |  |  |  |
|  | Unadjusted |  | Ref. | 1 (-16, 22) | -1 (-18, 20) | 0.94 |
|  | Model I** |  | Ref. | 3 (-15, 25) | -4 (-21, 17) | 0.70 |
|  | Model II*** |  | Ref. | 7 (-12, 31) | -1 (-19, 22) | 0.97 |
| IGF-1 | |  |  |  |  |  |
|  | Unadjusted |  | Ref. | -3 (-12, 8) | -4 (-13, 7) | 0.48 |
|  | Model I** |  | Ref. | -3 (-13, 8) | -5 (-15, 5) | 0.30 |
|  | Model II*** |  | Ref. | -3 (-13, 8) | -4 (-14, 7) | 0.46 |
| IGFBP-3 | |  |  |  |  |  |
|  | Unadjusted |  | Ref. | -3 (-10, 4) | 0 (-7, 8) | 0.93 |
|  | Model I** |  | Ref. | -1 (-8, 6) | 0 (-7, 7) | 0.98 |
|  | Model II*** |  | Ref. | -3 (-9, 4) | 1 (-6, 9) | 0.75 |
| *p-value < 0.05, percentage difference was calculated as 100*(exp(β)-1), **adjusted for time of blood sampling (continuous variable), maternal smoking (yes/no), gestational age at blood sampling (continuous variable)), ***further adjusted for smoking status of son (daily smoker/occasional smoker/non-smoker), body fat percentage (continuous variable), weekly alcohol intake (continuous variable) and physical fitness (Good or very good/moderate/poor), Abbreviations: SHBG, sex hormone-binding globulin; LH, luteinizing hormone; INSL3, Insulin-like factor 3; FSH, follicle stimulating hormone; IGF-1, insulin-like growth factor 1; IGFBP-3, insulin-like growth factor-binding protein 3. | | | | | | |

| Supplementary table 3: Linear regression analysis of prenatal serum levels of 2-phenylphenol (2-PP) (tertiles) and reproductive hormones (back-transformed estimates to reflect percentage difference) in adulthood | | | | | | |
| --- | --- | --- | --- | --- | --- | --- |
|  | |  | Prenatal 2- phenylphenol (2-PP) levels | | |  |
|  | |  | Lowest tertile | Second tertile | Highest tertile | p-trend |
|  | |  |  |  |  |  |
| Total Testosterone | |  |  |  |  |  |
|  | Unadjusted |  | Ref. | -2 (-13, 10) | 11 (-1, 25) | 0.08 |
|  | Model I** |  | Ref. | -4 (-15, 8) | 8 (-4, 22) | 0.19 |
|  | Model II*** |  | Ref. | -5 (-16, 7) | 5 (-7, 19) | 0.44 |
| SHBG | |  |  |  |  |  |
|  | Unadjusted |  | Ref. | 7 (-9, 27) | 8 (-8, 28) | 0.34 |
|  | Model I** |  | Ref. | 4 (-12, 24) | 5 (-12, 25) | 0.58 |
|  | Model II*** |  | Ref. | 1 (-15, 18) | 0 (-16, 19) | 0.97 |
| Free Testosterone | |  |  |  |  |  |
|  | Unadjusted |  | Ref. | -5 (-15, 6) | 9 (-2, 22) | 0.14 |
|  | Model I** |  | Ref. | -6 (-16, 5) | 7 (-4, 20) | 0.21 |
|  | Model II*** |  | Ref. | -5 (-15, 6) | 7 (-5, 21) | 0.28 |
| Estradiol | |  |  |  |  |  |
|  | Unadjusted |  | Ref. | -3 (-14, 10) | 6 (-7, 20) | 0.38 |
|  | Model I** |  | Ref. | -3 (-15, 10) | -5 (-16, 8) | 0.37 |
|  | Model II*** |  | Ref. | -4 (-16, 10) | 6 (-8, 22) | 0.44 |
| LH | |  |  |  |  |  |
|  | Unadjusted |  | Ref. | 2 (-15, 23) | 8 (-10, 31) | 0.40 |
|  | Model I** |  | Ref. | 3 (-15, 25) | 10 (-10, 34) | 0.35 |
|  | Model II*** |  | Ref. | 1 (-17, 23) | 7 (-13, 33) | 0.51 |
| Total Testosterone/LH | |  |  |  |  |  |
|  | Unadjusted |  | Ref. | -4 (-22, 18) | 2 (-17, 26) | 0.82 |
|  | Model I** |  | Ref. | -7 (-25, 15) | -1 (-21, 22) | 0.90 |
|  | Model II*** |  | Ref. | -6 (-24, 16) | -2 (-22, 23) | 0.85 |
| INSL3 | |  |  |  |  |  |
|  | Unadjusted |  | Ref. | -16 (-31, 3) | -8 (-25, 13) | 0.44 |
|  | Model I** |  | Ref. | -17 (-33, 2) | -10 (-27, 12) | 0.36 |
|  | Model II*** |  | Ref. | -14 (-31, 7) | 7 (-26, 17) | 0.54 |
| FSH | |  |  |  |  |  |
|  | Unadjusted |  | Ref. | -13 (-35, 16) | -1 (-26, 32) | 0.95 |
|  | Model I** |  | Ref. | -14 (-36, 15) | -17 (-39, 14) | 0.90 |
|  | Model II*** |  | Ref. | -17 (-39, 14) | -6 (-32, 32) | 0.73 |
| Inhibin B | |  |  |  |  |  |
|  | Unadjusted |  | Ref. | -4 (-20, 16) | 1 (-16, 22) | 0.88 |
|  | Model I** |  | Ref. | -5 (-21, 15) | -1 (-18, 20) | 0.96 |
|  | Model II*** |  | Ref. | -6 (-23, 14) | -1 (-20, 21) | 0.89 |
| IGF-1 | |  |  |  |  |  |
|  | Unadjusted |  | Ref. | 1 (-9, 12) | 4 (-7, 15) | 0.49 |
|  | Model I** |  | Ref. | 0 (-10, 11) | 3 (-8, 14) | 0.60 |
|  | Model II*** |  | Ref. | -4 (-14, 6) | -1 (-11, 11) | 0.91 |
| IGFBP-3 | |  |  |  |  |  |
|  | Unadjusted |  | Ref. | -3 (-9, 4) | 2 (-5, 9) | 0.61 |
|  | Model I** |  | Ref. | -3 (-9, 5) | 2 (-5, 10) | 0.56 |
|  | Model II*** |  | Ref. | -4 (-11, 3) | 2 (-6, 9) | 0.69 |
| *p-value < 0.05, percentage difference was calculated as 100*(exp(β)-1), **adjusted for time of blood sampling (continuous variable), maternal smoking (yes/no), gestational age at blood sampling (continuous variable)), ***further adjusted for smoking status of son (daily smoker/occasional smoker/non-smoker), body fat percentage (continuous variable), weekly alcohol intake (continuous variable) and physical fitness (Good or very good/moderate/poor), Abbreviations: SHBG, sex hormone-binding globulin; LH, luteinizing hormone; INSL3, Insulin-like factor 3; FSH, follicle stimulating hormone; IGF-1, insulin-like growth factor 1; IGFBP-3, insulin-like growth factor-binding protein 3. | | | | | | |

| Supplementary table 4: Linear regression analysis of prenatal serum levels of Benzophenone-3 (BP-3) (tertiles) and reproductive hormones (back-transformed estimates to reflect percentage difference) in adulthood | | | | | | |
| --- | --- | --- | --- | --- | --- | --- |
|  | |  | Prenatal Benzophenone-3 (BP-3) levels | | |  |
|  | |  | Lowest tertile | Second tertile | Highest tertile | p-trend |
|  | |  |  |  |  |  |
| Total Testosterone | |  |  |  |  |  |
|  | Unadjusted |  | Ref. | 11 (-1, 25) | -1 (-12, 11) | 0.82 |
|  | Model I** |  | Ref. | 8 (-4, 22) | -4 (-15, 8) | 0.44 |
|  | Model II*** |  | Ref. | 16* (3, 31) | -1 (-12, 12) | 0.87 |
| SHBG | |  |  |  |  |  |
|  | Unadjusted |  | Ref. | 4 (-12, 23) | -12 (-25, 4) | 0.14 |
|  | Model I** |  | Ref. | 2 (-13, 21) | -14 (-28, 2) | 0.07 |
|  | Model II*** |  | Ref. | 12 (-5, 33) | -11 (-24, 6) | 0.18 |
| Free Testosterone | |  |  |  |  |  |
|  | Unadjusted |  | Ref. | 11 (-1, 24) | 6 (-5, 18) | 0.33 |
|  | Model I** |  | Ref. | 9 (-3, 23) | 4 (-8, 17) | 0.59 |
|  | Model II*** |  | Ref. | 13 (-1, 27) | 6 (-6, 19) | 0.39 |
| Estradiol | |  |  |  |  |  |
|  | Unadjusted |  | Ref. | 10 (-3, 24) | 8 (-5, 22) | 0.25 |
|  | Model I** |  | Ref. | 8 (-5, 23) | 7 (-72, 2) | 0.36 |
|  | Model II*** |  | Ref. | 7 (-7, 24) | 2 (-11, 18) | 0.78 |
| LH | |  |  |  |  |  |
|  | Unadjusted |  | Ref. | 17 (-3, 42) | 19 (-1, 43) | 0.07 |
|  | Model I** |  | Ref. | 19 (-2, 45) | 22 (-1, 48) | 0.06 |
|  | Model II*** |  | Ref. | 21 (-2, 49) | 18 (-4, 45) | 0.12 |
| Total Testosterone/LH | |  |  |  |  |  |
|  | Unadjusted |  | Ref. | -5 (-23, 16) | -17 (-32, 2) | 0.07 |
|  | Model I** |  | Ref. | -9 (-26, 13) | -21* (-36, -2) | 0.03 |
|  | Model II*** |  | Ref. | -4 (-23, 20) | -16 (-32, 5) | 0.12 |
| INSL3 | |  |  |  |  |  |
|  | Unadjusted |  | Ref. | 9 (-1, 33) | 21 (-1, 48) | 0.06 |
|  | Model I** |  | Ref. | 8 (-13, 34) | 19 (-4, 48) | 0.10 |
|  | Model II*** |  | Ref. | 3 (-18, 29) | 26* (1, 58) | 0.04 |
| FSH | |  |  |  |  |  |
|  | Unadjusted |  | Ref. | -13 (-35, 16) | -1 (-26, 31) | 0.93 |
|  | Model I** |  | Ref. | -15 (-37, 15) | -4 (-29, 31) | 0.85 |
|  | Model II*** |  | Ref. | -15 (-39, 19) | -5 (-32, 33) | 0.78 |
| Inhibin B | |  |  |  |  |  |
|  | Unadjusted |  | Ref. | 1 (-16, 21) | -13 (-28, 4) | 0.12 |
|  | Model I** |  | Ref. | 2 (-16, 23) | -13 (-29, 5) | 0.13 |
|  | Model II*** |  | Ref. | 5 (-15, 29) | -8 (-25, 12) | 0.39 |
| IGF-1 | |  |  |  |  |  |
|  | Unadjusted |  | Ref. | -3 (-13, 7) | 1 (-9, 12) | 0.84 |
|  | Model I** |  | Ref. | -3 (-13, 8) | 2 (-9, 13) | 0.76 |
|  | Model II*** |  | Ref. | -2 (-13, 9) | 0 (-11, 12) | 0.99 |
| IGFBP-3 | |  |  |  |  |  |
|  | Unadjusted |  | Ref. | 3 (-4, 11) | 2 (-5, 9) | 0.67 |
|  | Model I** |  | Ref. | 4 (-3, 12) | 3 (-4, 11) | 0.49 |
|  | Model II*** |  | Ref. | 4 (-3, 12) | 0 (-7, 7) | 0.95 |
| *p-value < 0.05, percentage difference was calculated as 100*(exp(β)-1), **adjusted for time of blood sampling (continuous variable), maternal smoking (yes/no), gestational age at blood sampling (continuous variable)), ***further adjusted for smoking status of son (daily smoker/occasional smoker/non-smoker), body fat percentage (continuous variable), weekly alcohol intake (continuous variable) and physical fitness (Good or very good/moderate/poor), Abbreviations: SHBG, sex hormone-binding globulin; LH, luteinizing hormone; INSL3, Insulin-like factor 3; FSH, follicle stimulating hormone; IGF-1, insulin-like growth factor 1; IGFBP-3, insulin-like growth factor-binding protein 3. | | | | | | |

| Supplementary table 5: Linear regression analysis of prenatal serum levels of 4-hydroxy-benzophenone (4-HBP) (tertiles) and reproductive hormones (back-transformed estimates to reflect percentage difference) in adulthood | | | | | | |
| --- | --- | --- | --- | --- | --- | --- |
|  | |  | Prenatal 4-hydroxy-benzophenone (4-HBP) levels | | |  |
|  | |  | Lowest tertile | Second tertile | Highest tertile | p-trend |
|  | |  |  |  |  |  |
| Total Testosterone | |  |  |  |  |  |
|  | Unadjusted |  | Ref. | 4 (8, 18) | 5 (-7, 18) | 0.42 |
|  | Model I** |  | Ref. | 3 (-9, 17) | 4 (-8, 17) | 0.55 |
|  | Model II*** |  | Ref. | 3 (-8, 17) | 5 (-7, 19) | 0.42 |
| SHBG | |  |  |  |  |  |
|  | Unadjusted |  | Ref. | 9 (-8, 30) | 4 (-12, 23) | 0.67 |
|  | Model I** |  | Ref. | 11 (-7, 31) | 4 (-12, 24) | 0.64 |
|  | Model II*** |  | Ref. | 12 (-5, 32) | 3 (-13, 21) | 0.79 |
| Free Testosterone | |  |  |  |  |  |
|  | Unadjusted |  | Ref. | 1 (-10, 13) | 4 (-7, 17) | 0.44 |
|  | Model I** |  | Ref. | -1 (-12, 11) | 3 (-9, 15) | 0.63 |
|  | Model II*** |  | Ref. | -1 (-12, 11) | 6 (-6, 19) | 0.35 |
| Estradiol | |  |  |  |  |  |
|  | Unadjusted |  | Ref. | 8 (-5, 22) | 6 (-6, 20) | 0.34 |
|  | Model I** |  | Ref. | 6 (-7, 21) | 5 (-8, 19) | 0.48 |
|  | Model II*** |  | Ref. | 5 (-9, 20) | -3 (-10, 18) | 0.70 |
| LH | |  |  |  |  |  |
|  | Unadjusted |  | Ref. | 3 (-15, 25) | -1 (-18, 20) | 0.93 |
|  | Model I** |  | Ref. | 3 (-16, 25) | -1 (-19, 20) | 0.87 |
|  | Model II*** |  | Ref. | 2 (-17, 25) | -5 (-22, 17) | 0.65 |
| Total Testosterone/LH | |  |  |  |  |  |
|  | Unadjusted |  | Ref. | 1 (-18, 25) | 6 (-14, 30) | 0.59 |
|  | Model I** |  | Ref. | 1 (-19, 25) | 5 (-15, 30) | 0.63 |
|  | Model II*** |  | Ref. | 2 (-18, 26) | 10 (-11, 37) | 0.37 |
| INSL3 | |  |  |  |  |  |
|  | Unadjusted |  | Ref. | 13 (-8, 39) | 21 (-1, 47) | 0.07 |
|  | Model I** |  | Ref. | 12 (-9, 38) | 20 (-2, 48) | 0.08 |
|  | Model II*** |  | Ref. | 13 (-9, 41) | 21 (-3, 51) | 0.09 |
| FSH | |  |  |  |  |  |
|  | Unadjusted |  | Ref. | 15 (-14, 53) | -3 (-27, 29) | 0.82 |
|  | Model I** |  | Ref. | 15 (-15, 55) | -3 (-28, 30) | 0.81 |
|  | Model II*** |  | Ref. | 13 (-19, 56) | 0 (-28, 39) | 0.99 |
| Inhibin B | |  |  |  |  |  |
|  | Unadjusted |  | Ref. | -14 (-28, 3) | -10 (-25, 8) | 0.25 |
|  | Model I** |  | Ref. | -13 (-28, 5) | -9 (-25, 10) | 0.34 |
|  | Model II*** |  | Ref. | -9 (-25, 11) | -9 (-25, 12) | 0.39 |
| IGF-1 | |  |  |  |  |  |
|  | Unadjusted |  | Ref. | -5 (-14, 5) | -9 (-17, 1) | 0.08 |
|  | Model I** |  | Ref. | -5 (-14, 6) | -8 (-17, 2) | 0.11 |
|  | Model II*** |  | Ref. | -5 (-15, 5) | -10 (-19, 1) | 0.06 |
| IGFBP-3 | |  |  |  |  |  |
|  | Unadjusted |  | Ref. | 0 (-7, 7) | -4 (-10, 3) | 0.28 |
|  | Model I** |  | Ref. | 1 (-6, 8) | -3 (-9, 4) | 0.42 |
|  | Model II*** |  | Ref. | -1 (-8, 7) | -5 (-12, 2) | 0.15 |
| *p-value < 0.05, percentage difference was calculated as 100*(exp(β)-1) **adjusted for time of blood sampling (continuous variable), maternal smoking (yes/no), gestational age at blood sampling (continuous variable)), ***further adjusted for smoking status of son (daily smoker/occasional smoker/non-smoker), body fat percentage (continuous variable), weekly alcohol intake (continuous variable) and physical fitness (Good or very good/moderate/poor), Abbreviations: SHBG, sex hormone-binding globulin; LH, luteinizing hormone; INSL3, Insulin-like factor 3; FSH, follicle stimulating hormone; IGF-1, insulin-like growth factor 1; IGFBP-3, insulin-like growth factor-binding protein 3. | | | | | | |

| Supplementary table 6: Linear regression analysis of prenatal serum levels of 4-methyl-benzophenone (tertiles) and reproductive hormones (back-transformed estimates to reflect percentage difference) in adulthood | | | | | | |
| --- | --- | --- | --- | --- | --- | --- |
|  | |  | Prenatal 4-methyl-benzophenone (4-MBP) levels | | |  |
|  | |  | Lowest tertile | Second tertile | Highest tertile | p-trend |
|  | |  |  |  |  |  |
| Total Testosterone | |  |  |  |  |  |
|  | Unadjusted |  | Ref. | 5 (-7, 18) | 8 (-4, 22) | 0.19 |
|  | Model I** |  | Ref. | 4 (-8, 17) | 6 (-6, 20) | 0.33 |
|  | Model II*** |  | Ref. | 3 (-9, 17) | 7 (-5, 22) | 0.16 |
| SHBG | |  |  |  |  |  |
|  | Unadjusted |  | Ref. | -5 (-19, 13) | 1 (-15, 20) | 0.91 |
|  | Model I** |  | Ref. | -6 (-20, 12) | -1 (-16, 17) | 0.91 |
|  | Model II*** |  | Ref. | 0 (-16, 19) | 2 (-14, 21) | 0.82 |
| Free Testosterone | |  |  |  |  |  |
|  | Unadjusted |  | Ref. | 9 (-2, 22) | 10 (-2, 23) | 0.10 |
|  | Model I** |  | Ref. | 8 (-4, 21) | 9 (-3, 22) | 0.16 |
|  | Model II*** |  | Ref. | 4 (-8, 18) | 9 (-4, 23) | 0.18 |
| Estradiol | |  |  |  |  |  |
|  | Unadjusted |  | Ref. | 0 (-12, 13) | 8 (-5, 22) | 0.22 |
|  | Model I** |  | Ref. | -1 (-12, 13) | 8 (-5, 22) | 0.25 |
|  | Model II*** |  | Ref. | -2 (-15, 12) | 3 (-11, 18) | 0.71 |
| LH | |  |  |  |  |  |
|  | Unadjusted |  | Ref. | 6 (-13, 28) | 3 (-15, 25) | 0.76 |
|  | Model I** |  | Ref. | 5 (-13, 28) | 3 (-16, 25) | 0.78 |
|  | Model II*** |  | Ref. | -2 (-21, 21) | -4 (-22, 19) | 0.71 |
| Total Testosterone/LH | |  |  |  |  |  |
|  | Unadjusted |  | Ref. | 0 (-19, 23) | 5 (-15, 30) | 0.64 |
|  | Model I** |  | Ref. | -2 (-20, 21) | 3 (-16, 28) | 0.76 |
|  | Model II*** |  | Ref. | 6 (-16, 32) | 12 (-10, 39) | 0.32 |
| INSL3 | |  |  |  |  |  |
|  | Unadjusted |  | Ref. | 1 (-18, 24) | -7 (-24, 14) | 0.47 |
|  | Model I** |  | Ref. | 1 (-18, 24) | -7 (-25, 14) | 0.48 |
|  | Model II*** |  | Ref. | -8 (-27, 16) | -9 (-27, 14) | 0.42 |
| FSH | |  |  |  |  |  |
|  | Unadjusted |  | Ref. | 12 (-16, 49) | -8 (-31, 23) | 0.59 |
|  | Model I** |  | Ref. | 12 (-16, 50) | -8 (-31, 24) | 0.60 |
|  | Model II*** |  | Ref. | 19 (-15, 66) | -9 (-34, 26) | 0.55 |
| Inhibin B | |  |  |  |  |  |
|  | Unadjusted |  | Ref. | -7 (-23, 12) | -1 (-17, 20) | 0.94 |
|  | Model I** |  | Ref. | -7 (-22, 13) | -1 (-18, 20) | 0.94 |
|  | Model II*** |  | Ref. | -7 (-24, 15) | 3 (-16, 26) | 0.75 |
| IGF-1 | |  |  |  |  |  |
|  | Unadjusted |  | Ref. | -4 (-13, 7) | 0 (-10, 11) | 0.97 |
|  | Model I** |  | Ref. | -4 (-13, 7) | 0 (-10, 11) | 0.99 |
|  | Model II*** |  | Ref. | -2 (-12, 10) | -1 (-12, 10) | 0.79 |
| IGFBP-3 | |  |  |  |  |  |
|  | Unadjusted |  | Ref. | 1 (-6, 8) | 1 (-6, 8) | 0.79 |
|  | Model I** |  | Ref. | 2 (-5, 9) | 2 (-5, 10) | 0.59 |
|  | Model II*** |  | Ref. | 3 (-5, 11) | 0 (-7, 8) | 0.94 |
| *p-value < 0.05, percentage difference was calculated as 100*(exp(β)-1), **adjusted for time of blood sampling (continuous variable), maternal smoking (yes/no), gestational age at blood sampling (continuous variable)), ***further adjusted for smoking status of son (daily smoker/occasional smoker/non-smoker), body fat percentage (continuous variable), weekly alcohol intake (continuous variable) and physical fitness (Good or very good/moderate/poor), Abbreviations: SHBG, sex hormone-binding globulin; LH, luteinizing hormone; INSL3, Insulin-like factor 3; FSH, follicle stimulating hormone; IGF-1, insulin-like growth factor 1; IGFBP-3, insulin-like growth factor-binding protein 3. | | | | | | |

| Supplementary table 7: Linear regression analysis of prenatal serum levels of bisphenol A (BPA) (tertiles) and semen parameters and anogenital distance in adulthood | | | | | | |
| --- | --- | --- | --- | --- | --- | --- |
|  | |  | Prenatal Bisphenol A (BPA) levels | | |  |
|  | |  | Lowest tertile | Second tertile | Highest tertile | p-trend |
|  | |  |  |  |  |  |
| Concentration (percentage difference) | |  |  |  |  |  |
|  | Unadjusted |  | Ref. | 13 (-38, 108) | 2 (-44, 87) | 0.95 |
|  | Model I** |  | Ref. | 16 (-38, 117) | 0 (-47, 88) | 0.99 |
|  | Model II*** |  | Ref. | 30 (-34, 154) | -5 (-52, 88) | 0.87 |
| Semen volume (percentage difference) | |  |  |  |  |  |
|  | Unadjusted |  | Ref. | 2 (-22, 33) | -1 (-25, 29) | 0.92 |
|  | Model I** |  | Ref. | 6 (-19, 39) | 4 (-20, 36) | 0.76 |
|  | Model II*** |  | Ref. | 19 (-8, 53) | 10 (-15, 42) | 0.48 |
| Total sperm count (percentage difference) | |  |  |  |  |  |
|  | Unadjusted |  | Ref. | 15 (-42, 129) | 1 (-49, 100) | 0.99 |
|  | Model I** |  | Ref. | 23 (-40, 151) | 4 (-49, 113) | 0.92 |
|  | Model II*** |  | Ref. | 54 (-26, 223) | 4 (-51, 120) | 0.92 |
| Morphology (untransformed) | |  |  |  |  |  |
|  | Unadjusted |  | Ref. | 0.1 (-0.2, 2.2) | 0.1 (-1.9, 2.2) | 0.90 |
|  | Model I** |  | Ref. | -0.2 (-2.3, 1.9) | -0.3 (-2.5, 1.8) | 0.75 |
|  | Model II*** |  | Ref. | 0.1 (-2.1, 2.3) | -0.5 (-2.8, 1.7) | 0.65 |
| Motility (untransformed) | |  |  |  |  |  |
|  | Unadjusted |  | Ref. | -2.5 (-10.8, 5.9) | 0.0 (-8.4, 8.3) | 0.99 |
|  | Model I** |  | Ref. | -5.0 (-13.4, 3.5) | -5.1 (-13.8, 3.5) | 0.24 |
|  | Model II**** |  | Ref. | -1.6 (-9.9, 6.8) | -3.6 (-12.2, 5.0) | 0.40 |
| AGD_AS_ (percentage difference) | |  |  |  |  |  |
|  | Unadjusted |  | Ref. | 9 (-4, 24) | -4 (-16, 8) | 0.48 |
|  | Model I** |  | Ref. | 10 (-3, 24) | -5 (-16, 7) | 0.43 |
|  | Model II*** |  | Ref. | 11 (-3, 26) | -8 (-20, 6) | 0.34 |
| AGD_AP_ (percentage difference) | |  |  |  |  |  |
|  | Unadjusted |  | Ref. | 2 (-2, 7) | -2 (-7, 2) | 0.26 |
|  | Model I** |  | Ref. | 3 (-1, 8) | -2 (-6, 3) | 0.49 |
|  | Model II*** |  | Ref. | 4 (-1, 8) | -1 (-6, 3) | 0.68 |
| *p-value < 0.05, percentage difference was calculated as 100*(exp(β)-1)**adjusted for time of abstinence (continuous variable), maternal smoking (yes/no), gestational age at blood sampling (continuous variable), ***further adjusted for smoking status of son (daily smoker/occasional smoker/non-smoker), body fat percentage (continuous variable), weekly alcohol intake (continuous variable) and physical fitness (Good or very good/moderate/poor), *** further adjusted for time between delivery of semen sample and start of motility analysis (continuous variable). Abbreviations: AGDas, anogenital distance from anus to scrotum; AGDap, anogenital distance from anus to penis. | | | | | | |

| Supplementary table 8: Linear regression analysis of prenatal serum levels of triclosan (TCS) (tertiles) and semen parameters and anogenital distance in adulthood | | | | | | |
| --- | --- | --- | --- | --- | --- | --- |
|  | |  | Prenatal Triclosan (TCS) levels | | |  |
|  | |  | Lowest tertile | Second tertile | Highest tertile | p-trend |
|  | |  |  |  |  |  |
| Concentration (percentage difference) | |  |  |  |  |  |
|  | Unadjusted |  | Ref. | 9 (-40, 99) | -33 (-63, 22) | 0.18 |
|  | Model I** |  | Ref. | 4 (-44, 94) | -36 (-66, 21) | 0.16 |
|  | Model II*** |  | Ref. | 10 (-43, 111) | -36 (-68, 27) | 0.21 |
| Semen volume (percentage difference) | |  |  |  |  |  |
|  | Unadjusted |  | Ref. | 22 (-7, 60) | 12 (-14, 47) | 0.43 |
|  | Model I** |  | Ref. | 24 (-5, 61) | 14 (-13, 49) | 0.36 |
|  | Model II*** |  | Ref. | 16 (-9, 49) | 25 (-4, 62) | 0.09 |
| Total sperm count (percentage difference) | | |  |  |  |  |
|  | Unadjusted |  | Ref. | 33 (-33, 164) | -25 (-62, 48) | 0.38 |
|  | Model I** |  | Ref. | 29 (-36, 160) | -27 (-64, 50) | 0.38 |
|  | Model II*** |  | Ref. | 28 (-38, 163) | -20 (-63, 70) | 0.58 |
| Morphology (untransformed) | |  |  |  |  |  |
|  | Unadjusted |  | Ref. | -0.7 (-2.7, 1.4) | 0.1 (-2.0, 2.1) | 0.95 |
|  | Model I** |  | Ref. | -0.6 (-2.7, 1.5) | -0.1 (-2.3, 2.0) | 0.93 |
|  | Model II*** |  | Ref. | -0.9 (-3.0, 1.3) | 0.7 (-1.6, 2.9) | 0.61 |
| Motility (untransformed) | |  |  |  |  |  |
|  | Unadjusted |  | Ref. | 1.2 (-7.2, 9.6) | 2.2 (-6.2, 10.7) | 0.60 |
|  | Model I** |  | Ref. | 3.4 (-5.1, 12.0) | 1.4 (-7.5, 10.3) | 0.75 |
|  | Model II**** |  | Ref. | 2.2 (-6.1, 10.4) | 1.6 (-7.3, 10.5) | 0.71 |
| AGD_AS_ (percentage difference) | |  |  |  |  |  |
|  | Unadjusted |  | Ref. | 2 (-10, 16) | 0 (-13, 14) | 0.96 |
|  | Model I** |  | Ref. | 0 (-11, 14) | -1 (13, 12) | 0.84 |
|  | Model II*** |  | Ref. | 0 (-13, 15) | -2 (-15, 13) | 0.74 |
| AGD_AP_ (percentage difference) | |  |  |  |  |  |
|  | Unadjusted |  | Ref. | -1 (-5, 3) | -3 (-8, 1) | 0.12 |
|  | Model I** |  | Ref. | -1 (-6, 3) | -3 (-7, 2) | 0.18 |
|  | Model II*** |  | Ref. | -2 (-7, 2) | -3 (-7, 2) | 0.24 |
| *p-value < 0.05, percentage difference was calculated as 100*(exp(β)-1), **adjusted for time of abstinence (continuous variable), maternal smoking (yes/no), gestational age at blood sampling (continuous variable), ***further adjusted for smoking status of son (daily smoker/occasional smoker/non-smoker), body fat percentage (continuous variable), weekly alcohol intake (continuous variable) and physical fitness (Good or very good/moderate/poor), *** further adjusted for time between delivery of semen sample and start of motility analysis (continuous variable). Abbreviations: AGDas, anogenital distance from anus to scrotum; AGDap, anogenital distance from anus to penis. | | | | | | |

| Supplementary table 9: Linear regression analysis of prenatal serum levels of 2-phenylphenol (2-PP) (tertiles) and semen parameters and anogenital distance in adulthood | | | | | | |
| --- | --- | --- | --- | --- | --- | --- |
|  | |  | Prenatal 2- phenylphenol (2-PP) levels | | |  |
|  | |  | Lowest tertile | Second tertile | Highest tertile | p-trend |
|  | |  |  |  |  |  |
| Concentration (percentage difference) | |  |  |  |  |  |
|  | Unadjusted |  | Ref. | 47 (-19, 166) | 80 (-1, 288) | 0.05 |
|  | Model I** |  | Ref. | 42 (-23, 163) | 74 (-7, 225) | 0.08 |
|  | Model II*** |  | Ref. | 42 (-27, 174) | 63 (-19, 228) | 0.17 |
| Semen volume (percentage difference) | |  |  |  |  |  |
|  | Unadjusted |  | Ref. | 4 (-20, 35) | 20 (-8, 58) | 0.18 |
|  | Model I** |  | Ref. | 1 (-22, 32) | 23 (-6, 60) | 0.13 |
|  | Model II*** |  | Ref. | 5 (-18, 35) | 15 (-12, 50) | 0.30 |
| Total sperm count (percentage difference) | |  |  |  |  |  |
|  | Unadjusted |  | Ref. | 53 (-21, 197) | 117* (11, 325) | 0.02 |
|  | Model I** |  | Ref. | 44 (-28, 187) | 113* (6, 329) | 0.03 |
|  | Model II*** |  | Ref. | 49 (-28, 207) | 87 (-13, 303) | 0.11 |
| Morphology (untransformed) | |  |  |  |  |  |
|  | Unadjusted |  | Ref. | 1.5 (-0.6, 3.5) | 1.7 (-0.4, 3.7) | 0.11 |
|  | Model I** |  | Ref. | 1.2 (-0.9, 3.3) | 1.4 (-0.8, 3.5) | 0.21 |
|  | Model II*** |  | Ref. | 1.4 (-0.8, 3.5) | 1.3 (-1.0, 3.6) | 0.26 |
| Motility (untransformed) | |  |  |  |  |  |
|  | Unadjusted |  | Ref. | 3.3 (-4.8, 11.4) | 9.9* (1.7, 18.1) | 0.02 |
|  | Model I** |  | Ref. | 4.0 (-4.4, 12.4) | 6.4 (-2.3, 15.1) | 0.14 |
|  | Model II**** |  | Ref. | 4.8 (-3.3, 12.8) | 3.0 (-5.7, 11.7) | 0.46 |
| AGD_AS_ (percentage difference) | |  |  |  |  |  |
|  | Unadjusted |  | Ref. | 8 (-5, 23) | 2 (-10, 16) | 0.73 |
|  | Model I** |  | Ref. | 5 (-7, 19) | -1 (-12, 12) | 0.90 |
|  | Model II*** |  | Ref. | 5 (-8, 21) | 0 (-13, 15) | 0.99 |
| AGD_AP_ (percentage difference) | |  |  |  |  |  |
|  | Unadjusted |  | Ref. | 0 (-4, 4) | 1 (-3, 6) | 0.53 |
|  | Model I** |  | Ref. | 0 (-5, 4) | 1 (-3, 6) | 0.62 |
|  | Model II*** |  | Ref. | 1 (-4, 5) | 3 (-2, 7) | 0.23 |
| *p-value < 0.05, percentage difference was calculated as 100*(exp(β)-1), **adjusted for time of abstinence (continuous variable), maternal smoking (yes/no), gestational age at blood sampling (continuous variable), ***further adjusted for smoking status of son (daily smoker/occasional smoker/non-smoker), body fat percentage (continuous variable), weekly alcohol intake (continuous variable) and physical fitness (Good or very good/moderate/poor), *** further adjusted for time between delivery of semen sample and start of motility analysis (continuous variable). Abbreviations: AGDas, anogenital distance from anus to scrotum; AGDap, anogenital distance from anus to penis. | | | | | | |

| Supplementary table 10: Linear regression analysis of prenatal serum levels of benzophenone-3 (tertiles) and semen parameters and anogenital distance in adulthood | | | | | | |
| --- | --- | --- | --- | --- | --- | --- |
|  | |  | Prenatal Benzophenone-3 (BP-3) levels | | |  |
|  | |  | Lowest tertile | Second tertile | Highest tertile | p-trend |
|  | |  |  |  |  |  |
| Concentration (percentage difference) | |  |  |  |  |  |
|  | Unadjusted |  | Ref. | 35 (-26, 147) | -10 (-55, 46) | 0.47 |
|  | Model I** |  | Ref. | 28 (-32, 142) | -25 (-60, 41) | 0.34 |
|  | Model II*** |  | Ref. | 41 (-30, 182) | -25 (-62, 48) | 0.39 |
| Semen volume (percentage difference) | |  |  |  |  |  |
|  | Unadjusted |  | Ref. | -6 (-28, 23) | 7 (-18, 40) | 0.62 |
|  | Model I** |  | Ref. | -4 (-27, 25) | 7 (-18, 40) | 0.61 |
|  | Model II*** |  | Ref. | 7 (-19,39) | 12 (-14, 45) | 0.41 |
| Total sperm count (percentage difference) | |  |  |  |  |  |
|  | Unadjusted |  | Ref. | 27 (-36, 153) | -14 (-56, 70) | 0.66 |
|  | Model I** |  | Ref. | 23 (-40, 153) | -20 (-61, 64) | 0.51 |
|  | Model II*** |  | Ref. | 50 (-30, 224) | -16 (-60, 78) | 0.62 |
| Morphology (untransformed) | |  |  |  |  |  |
|  | Unadjusted |  | Ref. | 1.3 (-0.7, 3.4) | 0.5 (-1.6, 2.5) | 0.65 |
|  | Model I** |  | Ref. | 1.6 (-0.5, 3.7) | 0.5 (-1.6, 2.7) | 0.67 |
|  | Model II*** |  | Ref. | 2.3* (0.1, 4.5) | 0.4 (-1.8, 2.6) | 0.77 |
| Motility (untransformed) | |  |  |  |  |  |
|  | Unadjusted |  | Ref. | 3.5 (-4.9, 11.9) | 1.6 (-6.7, 9.9) | 0.71 |
|  | Model I** |  | Ref. | 3.9 (-5.2, 13.0) | 3.7 (-5.2, 12.5) | 0.43 |
|  | Model II**** |  | Ref. | 5.3 (-3.6, 14.3) | 3.1 (-5.5, 11.7) | 0.49 |
| AGD_AS_ (percentage difference) | |  |  |  |  |  |
|  | Unadjusted |  | Ref. | 8 (-5, 23) | 13 (-1, 29) | 0.05 |
|  | Model I** |  | Ref. | 6 (-7, 20) | 10 (-3, 25) | 0.13 |
|  | Model II*** |  | Ref. | 5 (-9, 21) | 9 (-5, 26) | 0.22 |
| AGD_AP_ (percentage difference) | |  |  |  |  |  |
|  | Unadjusted |  | Ref. | 0 (-5, 4) | 3 (-2, 7) | 0.25 |
|  | Model I** |  | Ref. | -1 (-5, 3) | 1 (-3, 6) | 0.50 |
|  | Model II*** |  | Ref. | -2 (-6, 3) | -1 (-5, 4) | 0.74 |
| *p-value < 0.05, percentage difference was calculated as 100*(exp(β)-1), **adjusted for time of abstinence (continuous variable), maternal smoking (yes/no), gestational age at blood sampling (continuous variable), ***further adjusted for smoking status of son (daily smoker/occasional smoker/non-smoker), body fat percentage (continuous variable), weekly alcohol intake (continuous variable) and physical fitness (Good or very good/moderate/poor), *** further adjusted for time between delivery of semen sample and start of motility analysis (continuous variable). Abbreviations: AGDas, anogenital distance from anus to scrotum; AGDap, anogenital distance from anus to penis. | | | | | | |

| Supplementary table 11: Linear regression analysis of prenatal serum levels of 4-hydroxy-benzophenone (4-HBP) (tertiles) and semen parameters and anogenital distance in adulthood | | | | | | |
| --- | --- | --- | --- | --- | --- | --- |
|  | |  | Prenatal 4-hydroxy-benzophenone (4-HBP) levels | | |  |
|  | |  | Lowest tertile | Second tertile | Highest tertile | p-trend |
|  | |  |  |  |  |  |
| Concentration (percentage difference) | |  |  |  |  |  |
|  | Unadjusted |  | Ref. | -14 (-53, 59) | -23 (-58, 40) | 0.38 |
|  | Model I** |  | Ref. | -17 (-56, 57) | -27 (-61, 37) | 0.33 |
|  | Model II*** |  | Ref. | -17 (-58, 61) | -31 (-65, 36) | 0.28 |
| Semen volume (percentage difference) | |  |  |  |  |  |
|  | Unadjusted |  | Ref. | -13 (-33, 14) | -17 (-37, 8) | 0.16 |
|  | Model I** |  | Ref. | -17 (-36, 9) | -17 (-36, 8) | 0.17 |
|  | Model II*** |  | Ref. | -20 (-38, 3) | -17 (-36, 7) | 0.16 |
| Total sperm count (percentage difference) | |  |  |  |  |  |
|  | Unadjusted |  | Ref. | -25 (-62, 49) | -37 (-68, 25) | 0.19 |
|  | Model I** |  | Ref. | -30 (-66, 42) | -39 (-70, 23) | 0.16 |
|  | Model II*** |  | Ref. | -34 (-68, 37) | -43 (-73, 21) | 0.14 |
| Morphology (untransformed) | |  |  |  |  |  |
|  | Unadjusted |  | Ref. | 0.5 (-1.6, 2.6) | 0.5 (-1.6, 2.6) | 0.62 |
|  | Model I** |  | Ref. | 0.7 (-1.5, 2.8) | 0.8 (-1.3, 2.9) | 0.47 |
|  | Model II*** |  | Ref. | 0.6 (-1.6, 2.7) | 0.8 (-1.4, 3.1) | 0.46 |
| Motility (untransformed) | |  |  |  |  |  |
|  | Unadjusted |  | Ref. | 1.5 (-6.9, 9.8) | -2.8 (-11.2, 5.6) | 0.50 |
|  | Model I** |  | Ref. | 1.9 (-7.0, 10.7) | 0.1 (-9.0, 9.2) | 0.99 |
|  | Model II**** |  | Ref. | -0.4 (-8.8, 8.0) | -1.8 (-10.9, 7.3) | 0.69 |
| AGD_AS_ (percentage difference) | |  |  |  |  |  |
|  | Unadjusted |  | Ref. | 1 (-11, 16) | 1 (-12, 15) | 0.91 |
|  | Model I** |  | Ref. | 0 (-13, 14) | -2 (-14, 12) | 0.76 |
|  | Model II*** |  | Ref. | -1 (-14, 14) | -1 (-14, 14) | 0.94 |
| AGD_AP_ (percentage difference) | |  |  |  |  |  |
|  | Unadjusted |  | Ref. | 3 (-2, 7) | 0 (-4, 4) | 0.86 |
|  | Model I** |  | Ref. | 2 (-3, 7) | -1 (-6, 3) | 0.50 |
|  | Model II*** |  | Ref. | 2 (-3, 6) | -2 (-6, 3) | 0.42 |
| *p-value < 0.05, percentage difference was calculated as 100*(exp(β)-1), **adjusted for time of abstinence (continuous variable), maternal smoking (yes/no), gestational age at blood sampling (continuous variable), ***further adjusted for smoking status of son (daily smoker/occasional smoker/non-smoker), body fat percentage (continuous variable), weekly alcohol intake (continuous variable) and physical fitness (Good or very good/moderate/poor), *** further adjusted for time between delivery of semen sample and start of motility analysis (continuous variable). Abbreviations: AGDas, anogenital distance from anus to scrotum; AGDap, anogenital distance from anus to penis. | | | | | | |

| Supplementary table 12: Linear regression analysis of prenatal serum levels of 4-methyl-benzophenone (4-MBP) (tertiles) and semen parameters and anogenital distance in adulthood | | | | | | |
| --- | --- | --- | --- | --- | --- | --- |
|  | |  | Prenatal 4-methyl-benzophenone (4-MBP) levels | | |  |
|  | |  | Lowest tertile | Second tertile | Highest tertile | p-trend |
|  | |  |  |  |  |  |
| Concentration (percentage difference) | |  |  |  |  |  |
|  | Unadjusted |  | Ref. | 26 (-31, 131) | 44 (-21, 161) | 0.23 |
|  | Model I** |  | Ref. | 26 (-32, 136) | 41 (-25, 166) | 0.28 |
|  | Model II*** |  | Ref. | 8 (-47, 119) | 31 (-34, 164) | 0.43 |
| Semen volume (percentage difference) | |  |  |  |  |  |
|  | Unadjusted |  | Ref. | -12 (-33, 15) | -7 (-29, 22) | 0.62 |
|  | Model I** |  | Ref. | -10 (-31, 18) | -1 (-24, 30) | 0.96 |
|  | Model II*** |  | Ref. | -16 (-36, 10) | -7 (-29, 21) | 0.61 |
| Total sperm count (percentage difference) | |  |  |  |  |  |
|  | Unadjusted |  | Ref. | 11 (-44, 119) | 34 (-32, 166) | 0.39 |
|  | Model I** |  | Ref. | 14 (-44, 131) | 40 (-32, 187) | 0.35 |
|  | Model II*** |  | Ref. | -9 (-58, 98) | 22 (-43, 163) | 0.59 |
| Morphology (untransformed) | |  |  |  |  |  |
|  | Unadjusted |  | Ref. | 0.2 (-1.9, 2.2) | 1.5 (-0.5, 3.6) | 0.14 |
|  | Model I** |  | Ref. | -0.1 (-2.2, 2.0) | 1.2 (-0.9, 3.3) | 0.26 |
|  | Model II*** |  | Ref. | -1.0 (-3.3, 1.3) | 0.8 (-1.5, 3.0) | 0.46 |
| Motility (untransformed) | |  |  |  |  |  |
|  | Unadjusted |  | Ref. | 0.0 (-0.2, 0.2) | -0.1 (-0.3, 0.1) | 0.47 |
|  | Model I** |  | Ref. | 8.6* (0.5, 16.8) | 7.9 (-0.2, 16.1) | 0.06 |
|  | Model II**** |  | Ref. | 7.4 (-1.1, 15.9) | 4.5 (-4.1, 13.1) | 0.33 |
| AGD_AS_ (percentage difference) | |  |  |  |  |  |
|  | Unadjusted |  | Ref. | 6 (-7, 21) | -3 (-15, 10) | 0.61 |
|  | Model I** |  | Ref. | 3 (-9, 17) | -6 (-17, 6) | 0.31 |
|  | Model II*** |  | Ref. | 4 (-10, 20) | -6 (-18,7) | 0.30 |
| AGD_AP_ (percentage difference) | |  |  |  |  |  |
|  | Unadjusted |  | Ref. | 4 (-1, 8) | 0 (-5, 4) | 0.81 |
|  | Model I** |  | Ref. | 2 (-2, 7) | -1 (-5, 3) | 0.61 |
|  | Model II*** |  | Ref. | 1 (-3, 6) | -2 (-6, 2) | 0.28 |
| *p-value < 0.05, percentage difference was calculated as 100*(exp(β)-1), **adjusted for time of abstinence (continuous variable), maternal smoking (yes/no), gestational age at blood sampling (continuous variable), ***further adjusted for smoking status of son (daily smoker/occasional smoker/non-smoker), body fat percentage (continuous variable), weekly alcohol intake (continuous variable) and physical fitness (Good or very good/moderate/poor), *** further adjusted for time between delivery of semen sample and start of motility analysis (continuous variable). Abbreviations: AGDas, anogenital distance from anus to scrotum; AGDap, anogenital distance from anus to penis. | | | | | | |
